# Laparoscopic Resection of Incidentally Detected Early-Stage Synchronous Duodenal and Jejunal Gastrointestinal Stromal Tumors in a Patient with Neurofibromatosis Type 1

**DOI:** 10.70352/scrj.cr.25-0830

**Published:** 2026-04-18

**Authors:** Kazuhide Urabe, Akihiko Oshita, Daisuke Takei, Meguri Tanimoto, Yasutomo Ojima, Tetsuya Kagawa, Masaki Inoue, Shosuke Kitamura, Keiji Hanada, Shuji Yonehara, Shinya Takahashi, Yasunobu Tanimoto

**Affiliations:** 1 Department of Surgery, Hongo Central Hospital, Mihara, Hiroshima, Japan; 2 Department of Surgery, JA Onomichi General Hospital, Onomichi, Hiroshima, Japan; 3 Department of Gastroenterology, Hongo Central Hospital, Mihara, Hiroshima, Japan; 4 Department of Gastroenterology, JA Onomichi General Hospital, Onomichi, Hiroshima, Japan; 5 Department of Pathology, JA Onomichi General Hospital, Onomichi, Hiroshima, Japan; 6 Department of Surgery, Graduate School of Biomedical & Health Sciences, Hiroshima University, Hiroshima, Hiroshima, Japan

**Keywords:** gastrointestinal stromal tumors, laparoscopic surgery, screening, neurofibromatosis

## Abstract

**INTRODUCTION:**

Although gastrointestinal stromal tumors (GISTs) are more common in neurofibromatosis type 1 (NF1) than in sporadic disease, the role of screening has not been well established. This case report details the successful laparoscopic resection for duodenal and jejunal GISTs detected during a general health check-up in an asymptomatic early-stage patient.

**CASE PRESENTATION:**

A 47-year-old female was diagnosed with NF1 during childhood. Abdominal ultrasonography performed during a general health check-up revealed a mass near the liver, in the region extending from the duodenal bulb to the hepatoduodenal ligament. Contrast-enhanced CT revealed the presence of a second mass in the jejunum. No clinical symptoms or findings, such as anemia or obstruction, were observed. Laparoscopic surgery was performed for both diagnostic and therapeutic purposes. Based on intraoperative findings, enucleation was selected for the periduodenal tumor partially attached to the duodenal wall, and partial intestinal resection was performed for the jejunal lesion. Capsule rupture did not occur in either tumor. Histopathology confirmed a 2.5-cm duodenal GIST and a 3.5-cm jejunal GIST. Both tumors showed a mitotic count of <1 per 50 high-power fields and were classified as belonging to the low-risk group according to established recurrence risk systems. The patient is currently under surveillance with no recurrence observed 2 years and 7 months postoperatively.

**CONCLUSIONS:**

This case report describes an asymptomatic, early-stage duodenal and jejunal GISTs associated with NF1 that was successfully treated by minimally invasive laparoscopic surgery. Although routine screening is not currently recommended and a single case cannot establish its benefit, this case suggests a potential role for risk-based early detection strategies in selected patients.

## Abbreviations

CD34
: cluster of differentiation 34
CE-CT
: contrast-enhanced CT
c-kit
: KIT proto-oncogene receptor tyrosine kinase
DOG1
: discovered on GIST-1
EUS
: endoscopic ultrasonography
GIST
: gastrointestinal stromal tumor
NCCN
: National Comprehensive Cancer Network
NF1
: neurofibromatosis type 1

## INTRODUCTION

NF1, formerly known as von Recklinghausen’s disease, is an autosomal dominant disease characterized by café-au-lait spots and multiple subcutaneous neurofibromas of the skin. It is associated with a high incidence of systemic tumors.^1–4)^ Among these, GISTs have been reported in association with NF1.^5,6)^ NF1-associated GIST differ in characteristics from sporadic GIST; additionally, 60%–75% involve the small intestine (jejunum/ileum), and 43%–50% are multiple tumors.^5,6)^ GIST is the most common mesenchymal tumor of the gastrointestinal tract and is thought to arise from, or share phenotypic features with, interstitial cells of Cajal.^7,8)^ The prognosis of GIST correlates with tumor location, tumor size, and mitotic count. Prognostic classification based on these factors is crucial for determining treatment strategies.^7,9–11)^ A proportion of GISTs are detected incidentally. Approximately 18% of sporadic GISTs are asymptomatic at diagnosis,^12)^ whereas around 30% of NF1-associated GISTs may be detected without symptoms,^13)^ and the significance of screening for the early diagnosis of GIST in NF1 remains poorly discussed. We report a rare case of NF1 with synchronous duodenal and jejunal GISTs detected in an asymptomatic patient, both tumors being classified as belonging to the low-risk group based on several established recurrence risk classification systems,^9–11)^ and successfully treated by minimally invasive laparoscopic resection.

## CASE PRESENTATION

**Fig. 1:**
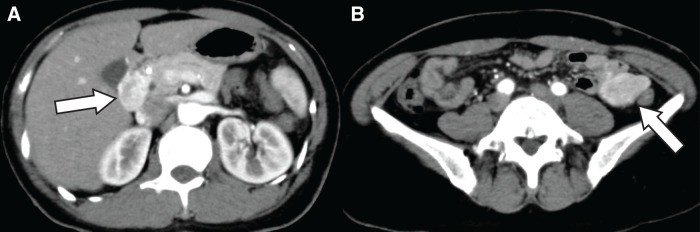
CE-CT findings. (**A**) A well-defined borderline mass 3.0 cm in size with clear contrast in the duodenal bulb near the pancreatic head (white arrow) is visible. (**B**) A mass 3.4 cm in size with a similar contrast effect is evident in the left lateral jejunum (white arrow). CE-CT, contrast enhanced-CT

**Fig. 2:**
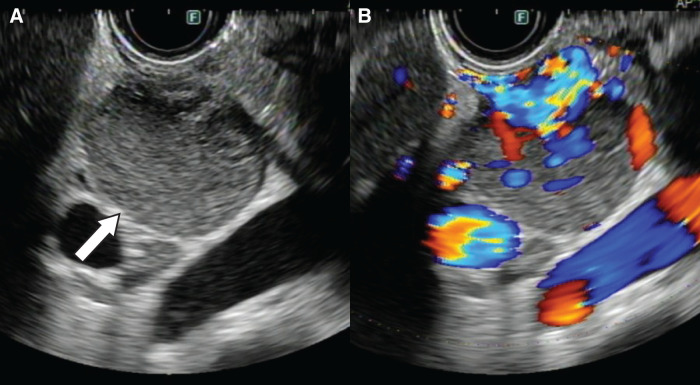
EUS findings. (**A**) A well-defined, homogeneous internal round tumor can be seen adjacent to the duodenal bulb (white arrow). (**B**) The tumor showed good blood flow on color Doppler. EUS, endoscopic ultrasonography

**Fig. 3:**
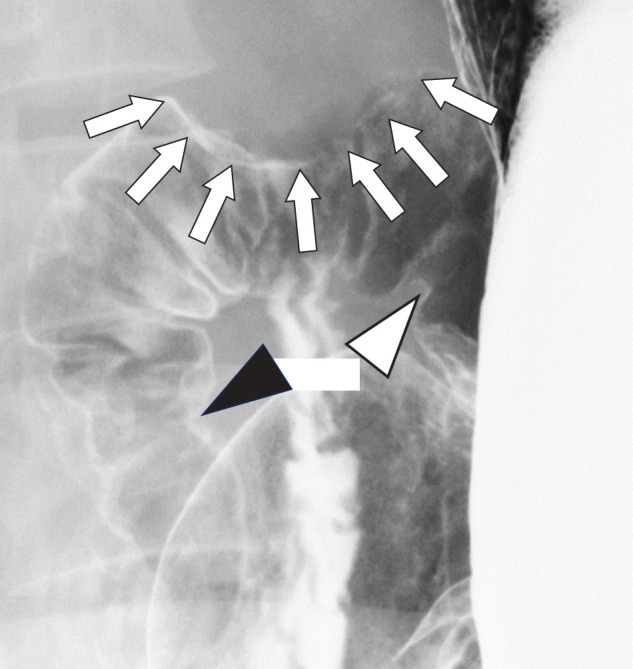
Hypotonic duodenography findings. Extramural compression of the duodenal bulb without mucosal changes is observed (arrows). The pyloric ring (white arrowhead) and duodenal papilla (black arrowhead) are identified. The lesion appears to arise from the wall opposite the papilla and is located away from the duodenal papilla.

A 47-year-old woman had been diagnosed with NF1 during elementary school based on the presence of café-au-lait spots and a subcutaneous schwannoma. She had discontinued regular follow-up in recent years because of relocation. During a general health checkup, abdominal ultrasonography incidentally detected a mass in the upper abdomen, located in the area corresponding to the liver, the duodenal bulb, or the hepatoduodenal ligament. The patient was asymptomatic. She had no family history of neurofibromatosis. Laboratory findings, including hemoglobin and tumor markers (carcinoembryonic antigen [CEA], carbohydrate antigen 19-9 [CA19-9], alpha-fetoprotein, CA125, and PIVKA2), were within normal ranges. Fecal occult blood tests performed on 2 occasions were negative. To further characterize the lesion, CE-CT was performed, which revealed a 3.0-cm, well-demarcated tumor with marked enhancement located from the duodenal bulb to the hepatoduodenal ligament (**Fig. 1A**). In addition, a 3.4-cm hyper-enhancing tumor was identified in the jejunum of the left abdomen (**Fig. 1B**). Subsequent upper and lower gastrointestinal endoscopy demonstrated no mucosal abnormalities in the duodenum or colon. To further evaluate the suspected lesions at the periduodenal or hepatoduodenal ligament sites, EUS was performed and revealed a well-perfused mass with marked intratumoral vascularity, in which continuity with the duodenal wall could not be clearly identified (**Fig. 2A**, **2B**). Although endoscopic US-guided fine-needle aspiration (EUS-FNA) was considered, it was not performed because color Doppler imaging demonstrated pronounced intratumoral vascularity, and the risk of hemorrhage was considered to be unacceptably high, thus outweighing the expected diagnostic benefit considering the uncertainty of obtaining a definitive histopathological diagnosis. Hypotonic duodenography was performed to further evaluate the anatomical relationship between the lesion and the duodenal lumen and to assist in tumor localization, revealing extramural compression of the duodenal bulb without mucosal changes. The lesion was located away from the duodenal papilla and appeared to arise from the wall opposite the papilla (**Fig. 3**). Capsule endoscopy was conducted to evaluate the small intestinal mucosa, including the jejunum, and revealed no mucosal abnormalities in the entire intestinal lumen. Finally, FDG-PET/CT was performed to assess the malignant potential of the peri-duodenal and small intestinal tumors and evaluate the presence of other gastrointestinal lesions, showing no abnormal uptake in either lesion and no evidence of distant disease.

Overall, endoscopy and endoscopic US demonstrated no apparent mucosal changes and no clear continuity between the tumor and the duodenal wall. Based on CE-CT, endoscopic US, and hypotonic duodenography, the lesion was preoperatively considered a non-epithelial tumor originating either from the duodenal bulb or the hepatoduodenal ligament. Although the exact layer of origin could not be determined, the absence of mucosal abnormalities on endoscopy suggested a deep location closer to the serosal side rather than the mucosa, making definitive classification as intramural or extramural difficult in the case that the lesion originated from the duodenal wall. The primary differential diagnoses included schwannoma and neurofibroma. In addition, given the background of NF1, duodenal GIST and neuroendocrine tumor were also considered in the differential diagnosis. The jejunal lesion was evaluated using the same diagnostic framework. As no imaging findings strongly suggested malignancy, laparoscopic surgery was planned as both a diagnostic and therapeutic approach.

**Fig. 4:**
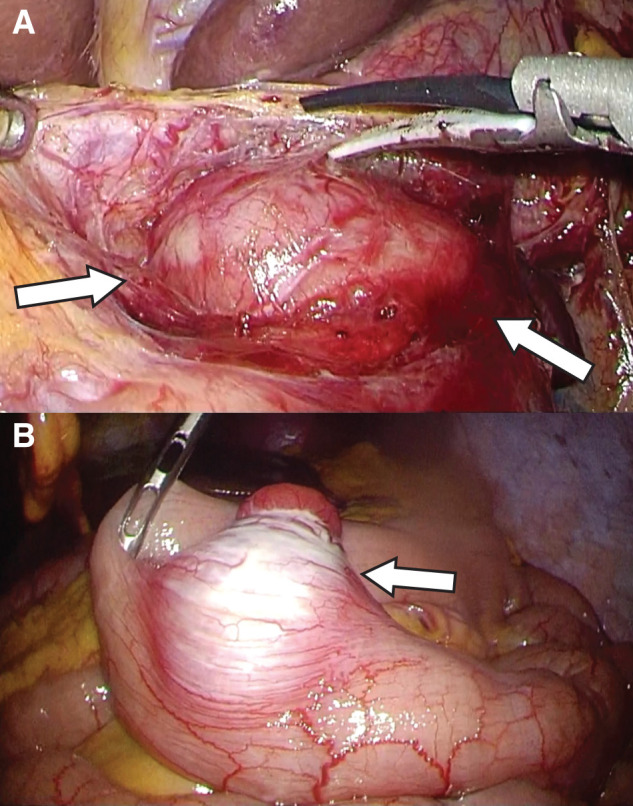
Intraoperative findings. (**A**) The tumor in the duodenal bulb was located outside the wall, contiguous to the duodenal wall. The duodenal lesion was resected locally without damage to the tumor capsule (white arrows). (**B**) A tumor expanding outside the wall in the jejunum was identified, and partial resection of the jejunum was performed (white arrow).

The operation was performed laparoscopically using a 5-port technique. Upon exploration of the abdominal cavity, a tumor located near the hepatoduodenal ligament was identified as an extraluminally protruding mass located adjacent to the cranial aspect of the duodenal bulb. The tumor was easily dissected from the gallbladder and hepatoduodenal ligament. Based on preoperative differential diagnosis and intraoperative findings, a benign neurogenic tumor, such as a schwannoma, was considered as a possible differential diagnosis. Preoperative imaging suggested that continuity between the tumor and the duodenal wall was limited to a very small area. Therefore, to minimize surgical invasiveness while ensuring diagnostic and therapeutic effectiveness, tumor enucleation was selected as a form of local resection. The tumor was carefully excised with partial attachment of the duodenal seromuscular layer, ensuring preservation of the tumor capsule and avoiding rupture (**Fig. 4A**). The duodenal mucosa was not opened. The resulting seromuscular defect was closed with hand-sewn sutures along the longitudinal axis of the duodenum to prevent postoperative stenosis and reinforced with an omental patch. The resected duodenal tumor was placed in a retrieval bag. Subsequently, the jejunal lesion was identified as a well-encapsulated extraluminal tumor protruding from the small intestinal wall in the left side abdominal space (**Fig. 4B**). An additional small incision was made at the umbilical port site, through which the jejunum was exteriorized. Partial jejunal resection was performed without capsule rupture, followed by hand-sewn layer-to-layer end-to-end anastomosis. Duodenal specimen was retrieved through the same mini-laparotomy. Total operative time was 3 hours and 13 minutes, and the estimated blood loss was 100 mL. No blood transfusion was required.

**Fig. 5:**
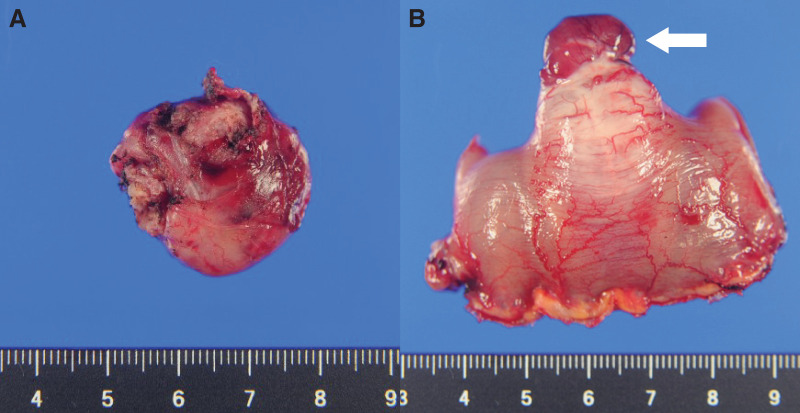
Macroscopic findings of the resected specimens. (**A**) The duodenal tumor shows a well-circumscribed nodular mass measuring 2.5 cm in diameter, extending to the serosal surface. (**B**) The jejunal tumor shows a nodular mass measuring 3.5 cm in diameter, extending to the serosa, with a gross appearance similar to the duodenal lesion (white arrow).

**Fig. 6:**
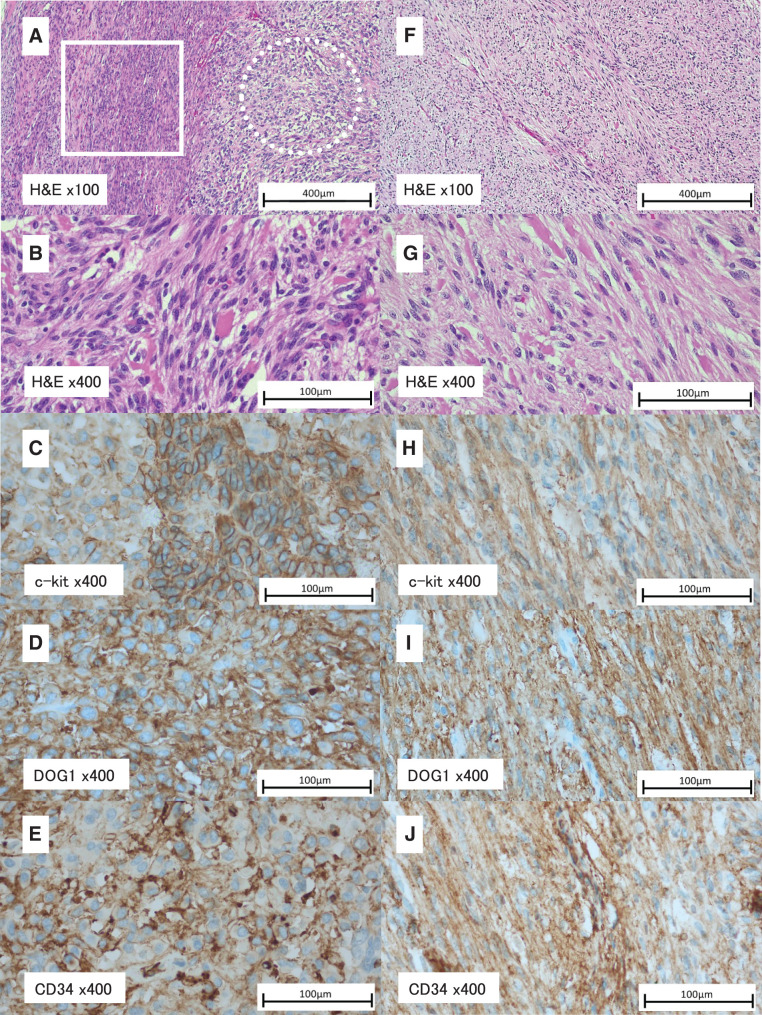
Histological and immunohistochemical findings. (**A**–**E**) Duodenal tumor. (**A**) Spindle-shaped tumor cells arranged in fascicles and bundles with extracellular collagen globules (skeinoid fibers) are observed (solid box). A focal area of epithelioid morphology composed of large polygonal cells is also noted (dotted circle). (**B**) Higher magnification view of the spindle-cell component (hematoxylin and eosin staining; **A**, ×100; **B**, ×400). (**C**) Tumor cells are positive for c-kit (×400). (**D**) Tumor cells are positive for DOG1 (×400). (**E**) Tumor cells are positive for CD34 (×400). (**F**–**J**) Jejunal tumor. (**F**, **G**) Spindle-shaped tumor cells arranged in fascicles and bundles with skeinoid fibers are observed, without nuclear pleomorphism (hematoxylin and eosin staining; **F**, ×100; **G**, ×400). (**H**) Tumor cells are positive for c-kit (×400). (**I**) Tumor cells are positive for DOG1 (×400). (**J**) Tumor cells are positive for CD34 (×400). CD34, cluster of differentiation 34; c-kit, KIT proto-oncogene receptor tyrosine kinase; DOG1, discovered on GIST-1; H&E, hematoxylin and eosin staining

Macroscopic examination of the resected duodenal specimen showed a well-circumscribed mass of 2.5 cm in diameter extending to the serosa (**Fig. 5A**). The cut surface showed a grey-white elastic hard mass in continuity with the duodenal muscularis propria. Capsular destruction was not seen. Neither hemorrhage nor necrosis was observed. Macroscopic examination of the resected jejunal specimen showed a nodular mass of 3.5 cm in diameter extending to the serosa (**Fig. 5B**). The cut surface showed a grey-white elastic hard mass in continuity to the mucosal surface with ulceration. Capsular destruction was not observed. On microscopic examination of the duodenal specimen, spindle shaped tumor cells were arranged in fascicles and bundles containing extracellular collagen globules (skeinoid fibers). Focal diffuse proliferation of large polygonal cells was noted, suggesting epithelioid morphology (**Fig. 6A**, **6B**). Nuclear pleomorphism was not observed. Immunophenotypically, both spindle shaped tumor cells and epithelioid tumor cells showed strong and diffuse expression of c-kit (**Fig. 6C**), DOG1 (**Fig. 6D**), and CD34 (**Fig. 6E**), appearing as cytoplasmic, membrane-associated staining. Moreover, they lacked S100 protein, desmin, and α-smooth muscle actin expression. Based on these findings, the duodenal tumor was diagnosed as a GIST, excluding schwannoma.

On microscopic examination of the jejunal specimen, spindle shaped tumor cells arranged in fascicles and bundles containing extracellular collagen globules (skeinoid fibers). Nuclear pleomorphism was not seen (**Fig. 6F**, **6G**). Immunophenotypically, spindle shaped tumor cells showed strong and diffuse expression of c-kit (**Fig. 6H**), DOG1 (**Fig. 6I**), and CD34 (**Fig. 6J**), which appeared as cytoplasmic, membrane associated staining. Additionally, they lacked S100 protein, desmin, and α-smooth muscle actin expression which are positive in schwannoma cells (no figure). Based on these findings, the jejunal tumor was also diagnosed as a GIST.

Both tumors were diagnosed as GIST, characterized by non-gastric lesions measuring 2.0–5.0 cm in diameter, with a mitotic count <5/50 HPF and no evidence of tumor rupture. Therefore, according to the major risk classification systems—NIH/Fletcher,^10)^ AFIP/Miettinen,^9)^ and modified NIH/Joensuu^11)^—the recurrence risk classifications of both tumors were diagnosed as belonging to the low-risk group.

The postoperative course was uneventful, and the patient discharged after 12 days from surgery. Postoperative surveillance included contrast-enhanced chest and abdominal CT at 6 months and 1 year after surgery. Following a contrast medium allergy at the 1-year examination, despite steroid premedication for multiple pre-existing drug allergies, follow-up was continued with non-contrast CT at 1 year 6 months, 2 years, and 2 years 6 months postoperatively. Upper gastrointestinal endoscopy targeting the duodenal resection site was performed at 1 and 2 years. The patient has been followed up monthly in the outpatient clinic. By 2 years and 7 months after surgery, no evidence of recurrence has been observed. Furthermore, after GIST treatment, the patient was referred to a dermatologist for NF1 management and has since maintained annual dermatological follow-up.

## DISCUSSION

We presented the case of a patient with NF1 who had a double GIST in the duodenum and jejunum. The patient was diagnosed when she was asymptomatic and underwent curative resection via minimally invasive surgery. This experience prompted reconsideration of the clinical significance of tumor detection screening in a patient with NF1.

Patients with NF1 have an increased overall tumor incidence of 22.8%, of which 9.1% are malignant, corresponding to a 2.7-fold increase in cancer risk compared with the general population.^2,4)^ Regarding the association between NF1 and GIST, GISTs have been reported to occur in 1.2%–6.3% of NF1 patients.^5,6,14)^ Conversely, NF1 has been identified in approximately 5% of patients with GISTs.^7)^ Collectively, these data indicate that GIST is an important gastrointestinal tumor associated with NF1.

Routine screening for GIST in asymptomatic NF1 patients is not currently recommended by major clinical guidelines.^15–18)^ Nevertheless, compared with sporadic GIST, NF1-associated GISTs are more likely to arise in the small intestine or duodenum rather than the stomach, to be multifocal, and to lack KIT or PDGFRA mutations, reflecting distinct molecular characteristics and consequently exhibiting limited responsiveness to imatinib.^5,7,19)^ In the present case, both tumors were classified as belonging to the low risk group and adjuvant therapy was not indicated; therefore, KIT/PDGFRA mutation analysis was not performed. Although mutation-related therapeutic implications cannot be discussed, early detection enabled curative resection using a minimally invasive approach, illustrating that selected NF1-associated GIST may be amenable to less invasive management when identified at an early stage.

Multiple modalities have been reported for detecting GIST, including fecal occult blood testing,^20)^ abdominal ultrasonography,^21)^ contrast-enhanced or non-contrast CT,^14,22,23)^ MRI,^24)^ FDG-PET/CT,^25)^ balloon-assisted enteroscopy,^26,27)^ and capsule endoscopy.^28)^ In this case, only contrast-enhanced CT identified both lesions. Although CT and MRI show reported sensitivities of 81.8%–89.2% and specificities of 89.3%–93.0%,^23,24)^ FDG-PET/CT has demonstrated a sensitivity of 89% and a specificity of 97%.^25)^ However, these data are largely derived from clinically apparent or advanced tumors larger than 5 cm in size; thus, diagnostic performance in small or early-stage GIST remains uncertain. A Japanese CT-based screening study identified GIST in 6 of 95 asymptomatic adult NF1 patients (6.3%), with tumor sizes ranging 1.8–6.0 cm and frequent multifocal involvement of the small intestine.^14)^

Sporadic GIST tends to present at 50–62 years of age, whereas NF1-associated GIST tends to present earlier (43.7–53 years).^7,9,13,20,29)^ European consensus recommendations have been incorporated into structured adult surveillance frameworks, which advise baseline evaluation at transition to adult care or at the first adult visit, followed by periodic clinical assessment and symptom-directed imaging.^30)^ Whole-body MRI may be considered to establish baseline tumor burden, and abdominal ultrasonography may be used according to clinical indication. Follow-up clinical examination is suggested at least annually in individuals younger than 50 years and every 2 years in those aged 50 years or older; routine interval imaging is not universally mandated.^16,30)^ Higher detection rates have also been reported in individuals with a family history of NF1.^31)^ Although this single case does not justify general screening, existing epidemiological data indicate that GIST can occur in NF1 without overt symptoms, warranting clinical attention. However, the optimal target population, imaging modality, and surveillance interval remain undefined, and any screening strategy should be approached cautiously, considering potential harms such as radiation exposure, false-positive findings, overdiagnosis, increased medical costs, and the need for prospective validation.

Regarding surgical procedure, according to the NCCN guidelines, surgical management of resectable GIST requires complete tumor removal with negative margins while avoiding capsule rupture; routine lymph node dissection is generally unnecessary, minimally invasive surgery may be considered depending on tumor characteristics, and careful intraoperative handling is recommended to minimize the risk of peritoneal dissemination.^32)^ However, the optimal surgical approach for duodenal GIST remains debated because of their anatomical complexity. In a retrospective analysis of duodenal GIST, local resection was associated with lower postoperative complication rates than procedures requiring gastrointestinal reconstruction depending on tumor location, whereas tumors measuring 5 cm or larger were linked to increased ICU admission, more postoperative complications, and longer hospital stays.^33)^ In another report, Dubois et al. conducted a multicenter European study of 100 patients with duodenal GIST, in which local resection—including enucleation—was evaluated in comparison with pancreatoduodenectomy.^34)^ In this study, enucleation, primarily performed for tumors smaller than 5 cm, demonstrated postoperative and long-term outcomes comparable to other forms of local resection. Compared with other local resections, enucleation was associated with a reported 5-year overall survival rate of 84% and no tumor recurrence. However, tumor rupture occurred in 7.7% of patients who underwent enucleation, whereas no tumor rupture was reported in the other surgical groups. These findings indicate that, although enucleation can be an effective surgical option for duodenal GIST in selected cases, careful patient selection and meticulous surgical technique are essential to minimize the risk of tumor rupture. In the present case, given the small tumor size and favorable intraoperative findings, laparoscopic enucleation was performed, and complete tumor removal without capsule rupture was achieved. From a retrospective perspective, this laparoscopic enucleation was consistent with the surgical strategies outlined in the NCCN guidelines and, in light of the previously reported evidence, may be considered an oncologically appropriate approach for duodenal GIST in cases such as that reported here.

For postoperative surveillance, current GIST guidelines do not provide specific prognostic classifications or standardized management strategies tailored to NF1-associated GIST.^18,32,35,36)^ However, NF1-associated GIST tends to present with multiple tumors either synchronously or metachronously, suggesting that prolonged follow-up may be considered to facilitate early detection of new or recurrent lesions. In the present case, dermatological follow-up for NF1 had been interrupted prior to presentation. Surgical treatment of NF1-associated GIST was performed by gastrointestinal surgeons who have continued GIST surveillance after intervention. This treatment also provided an opportunity to resume follow-up for NF1 by dermatologists, including surveillance for NF1-related complications, and coordinated multidisciplinary follow-up is currently ongoing.

## CONCLUSIONS

We report a rare case of asymptomatic, low-risk synchronous duodenal and jejunal GIST associated with NF1 that were successfully treated with minimally invasive laparoscopic surgery. While this single case cannot support recommendations for routine screening, it may provide preliminary insight into risk-based approaches to detection in selected patients.

## DECLARATIONS

### Use of Artificial Intelligence (AI) tools

Non-generative artificial intelligence tools, including DeepL and Grammarly, were used solely to assist with language editing, such as spelling and grammar. After drafting the manuscript, ChatGPT was used to improve the clarity and readability of selected English expressions. All AI-assisted outputs were carefully reviewed and edited by the authors, who take full responsibility for the accuracy and integrity of the content.
